# IRAMP: Investigation of Risk Assessment and Management Processes Using Datix Report Analysis and Observation of Clinical Team Meetings

**DOI:** 10.1192/bjo.2024.254

**Published:** 2024-08-01

**Authors:** Kay Sunderland, Emma Drysdale, Brian Gillatt, Alan Mackenzie, Paula McCahon

**Affiliations:** 1 NHS Greater Glasgow and Clyde, Glasgow, United Kingdom

## Abstract

**Aims:**

To investigate risk assessment and management processes across a health board in the context of the implementation of a new risk screening tool and risk policy using comparison of DATIX incidents before and after implementation of the CRAFT tool.

In mental health services, risk assessment and management are key responsibilities for clinical staff. A risk management tool that is structured and evidence-based aims to assist staff in managing risks including violence, self-harm, suicide and self-neglect.

It is not clear whether risk tools have clinical utility in influencing risk-related decision making and previous reviews within the health board indicated that risk policy was not being adhered to, prompting a review of the policy. Furthermore, policy recommends service user and carer collaboration with staff in all areas of mental health in Scotland but despite these recommendations there is little evidence to suggest they are routinely involved in risk assessment and management processes.

The present study is an opportunity to explore how teams think about and discuss risk management.

**Methods:**

We looked at data on patient incidents that occurred over 30 months from 1/1/19 to 30/09/21. The Datix data were subdivided into five main categories: Violence & Aggression, Challenging behaviour, self-harm, absconding/missing and Suicide.

**Results:**

Throughout the study period the category of Violence & Aggression was the most frequently reported Datix category for 28 out of 30 months, followed by Challenging Behaviour which was the second most frequent category for 22 out of 30 months and in the last year reports in this category have increased by 39.35%. The third most frequently reported category was self-harm and the fourth most reported category was Abscondment/Missing. The frequency of reports in this category increased over the study period.

The rate of suicide was consistently the lowest reported category and remained stable throughout the study period. With the exception of Violence and Aggression, all categories showed a general upwards trend in Datix report numbers.

**Conclusion:**

We have seen an increase in significant incidents in all categories reported using the DATIX system with the exception of suicide and violence and aggression during the study period. This suggests that further work is required to ascertain the reasons for this and what impact, if any, the change in CRAFT risk assessment tool has had.

